# Health, Wellbeing, and Prognosis of Australian Adolescents with Myalgic Encephalomyelitis/Chronic Fatigue Syndrome (ME/CFS): A Case-Controlled Follow-Up Study

**DOI:** 10.3390/jcm10163603

**Published:** 2021-08-16

**Authors:** Elisha K. Josev, Rebecca C. Cole, Adam Scheinberg, Katherine Rowe, Lionel Lubitz, Sarah J. Knight

**Affiliations:** 1 Neurodisability and Rehabilitation, Murdoch Children’s Research Institute, Royal Children’s Hospital, Melbourne 3052, Australia; beccole26@gmail.com (R.C.C.); adam.scheinberg@rch.org.au (A.S.); sarah.knight@mcri.edu.au (S.J.K.); 2 Department of Paediatrics, University of Melbourne, Melbourne 3052, Australia; 3 Department of Paediatrics, Monash University, Melbourne 3800, Australia; 4 Victorian Paediatric Rehabilitation Service, Royal Children’s Hospital, Melbourne 3052, Australia; 5 Department of General Medicine, Royal Children’s Hospital, Melbourne 3052, Australia; kathy@roweresearch.com (K.R.); lubitz@bigpond.net.au (L.L.)

**Keywords:** chronic fatigue syndrome, myalgic encephalomyelitis, follow-up, adolescence, health, wellbeing, diagnostic criteria

## Abstract

Background: The purpose of this study was to follow-up an Australian cohort of adolescents newly-diagnosed with ME/CFS at a tertiary paediatric ME/CFS clinic and healthy controls over a mean period of two years (range 1–5 years) from diagnosis. Objectives were to (a) examine changes over time in health and psychological wellbeing, (b) track ME/CFS symptomatology and fulfillment of paediatric ME/CFS diagnostic criteria over time, and (c) determine baseline predictors of ME/CFS criteria fulfilment at follow-up. Methods: 34 participants aged 13–18 years (25 ME/CFS, 23 controls) completed standardised questionnaires at diagnosis (baseline) and follow-up assessing fatigue, sleep quality and hygiene, pain, anxiety, depression, and health-related quality of life. ME/CFS symptomatology and diagnostic criteria fulfilment was also recorded. Results: ME/CFS patients showed significant improvement in most health and psychological wellbeing domains over time, compared with controls who remained relatively stable. However, fatigue, pain, and health-related quality of life remained significantly poorer amongst ME/CFS patients compared with controls at follow-up. Sixty-five percent of ME/CFS patients at baseline continued to fulfil ME/CFS diagnostic criteria at follow-up, with pain the most frequently experienced symptom. Eighty-two percent of patients at follow-up self-reported that they still had ME/CFS, with 79% of these patients fulfilling criteria. No significant baseline predictors of ME/CFS criteria fulfilment at follow-up were observed, although pain experienced at baseline was significantly associated with criteria fulfilment at follow-up (*R* = 0.6, *p* = 0.02). Conclusions: The majority of Australian adolescents with ME/CFS continue to fulfil diagnostic criteria at follow-up, with fatigue, pain, and health-related quality of life representing domains particularly relevant to perpetuation of ME/CFS symptoms in the early years following diagnosis. This has direct clinical impact for treating clinicians in providing a more realistic prognosis and highlighting the need for intervention with young people with ME/CFS at the initial diagnosis and start of treatment.

## 1. Introduction

Paediatric myalgic encephalomyelitis/chronic fatigue syndrome (ME/CFS) is a disabling condition of unknown etiology. It causes significant and well-documented adverse effects in physical and psychological functioning, school attendance and participation, and quality of life [1,2,3,4]. Less documented are the longer-term impacts on health and wellbeing for young people with ME/CFS in the years following diagnosis, in comparison with their healthy peers. Such information is invaluable for understanding illness course, prognosis, and potential targets for management and treatment of paediatric ME/CFS. It may also help identify potentially diverging developmental trajectories for patients with ME/CFS during adolescence and young adulthood; a period already characterised by considerable transition and change.

Significant change in emotional, social, hormonal, and physical functioning is typical in the transition from childhood to early adulthood [5]. Onset of paediatric ME/CFS during this period can therefore pose a diagnostic challenge. Indeed, paediatric ME/CFS is known to be associated with compromised physical health and psychological health and wellbeing, including greater fatigue, pain, anxiety and depressive symptoms, and poorer sleep quality and quality of life [6,7,8,9,10,11,12,13]. However, fatigue and insufficient, poor quality sleep is also prevalent amongst healthy high-school-aged Australians [14,15], rates of anxiety and depression tend to increase across mid to late adolescence [16,17,18], and health-related quality of life declines from 12 years of age onwards at a population level [19,20,21]. In order to quantify the impacts of paediatric ME/CFS on fatigue, sleep, emotional problems and health related quality of life, there is a need to compare the trajectories of these outcomes in both adolescents with ME/CFS and healthy adolescents using a longitudinal standardised design. Tracking specific ME/CFS symptomatology in the same patients over time using the same standardised measures has the benefit of identifying which illness aspects are endorsed most frequently (and perhaps, the ones that carry the most burden), as well as understanding the factors that might predict patients’ future wellbeing and health status.

While there are relatively few longitudinal studies assessing follow-up of adolescent patients with ME/CFS, the limited evidence available suggests that improvement and recovery are more likely in paediatric ME/CFS compared with adult ME/CFS [1]. To date, paediatric ME/CFS follow-up studies (ranging from 1 to 21 years follow-up) have reported recovery rates of between 5% and 83% [11,12,22,23,24,25,26,27,28,29,30], although there is variability in how recovery is defined across studies. Research has tended to use individuals’ self-defined recovery in the common domains of fatigue, physical functioning, and school attendance [30]. The latter measure may be problematic, however, for older adolescents who had already finished schooling at the follow-up time point, or for younger adolescents who are attending school but not functioning well due to ongoing cognitive disturbances.

Surprisingly few studies have focused on the health factors that may predict longer-term outcomes such as persistence of diagnostic symptoms, with studies tending to focus on risk factors for new-onset ME/CFS [15,31]. The few studies that have assessed these factors have been inconclusive. For example, studies of adolescents with ME/CFS-like symptoms have shown that baseline anxiety and depression predicts future fatigue persistence [32,33]. In contrast, subsequent studies have found no association between baseline depression and anxiety and recovery from paediatric ME/CFS at follow-up [11,26]. Other methodological factors have limited the ability to predict patients’ ME/CFS clinical status and symptom persistence at follow-up, such as the inclusion of patients whose baseline ME/CFS status was unable to be verified by a physician’s clinical diagnosis or did not fulfil diagnostic ME/CFS criteria, and/or the use of different tools or methods to measure patients’ symptoms at baseline and follow-up [12,28,34]. Understanding the relative importance of physical and psychological health factors to patients’ long-term outcome is, therefore, important for guiding future preventative, management and treatment approaches.

There were three mains aims for this study. First, we aimed to examine the change over time in factors associated with health and psychological wellbeing (i.e., fatigue, sleep quality and hygiene, pain, anxiety, depression, and HRQOL) in newly-diagnosed adolescents with ME/CFS relative to healthy adolescents, across a mean follow-up period of two years from diagnosis (and study enrolment). Second, we aimed to track patients’ ME/CFS symptomatology over time to determine the type and frequency of symptoms experienced, and fulfilment of paediatric ME/CFS diagnostic criteria at follow-up (i.e., prognosis). Finally, the third aim was to determine which aspects of health and psychological wellbeing at diagnosis best predicted ME/CFS criteria fulfilment at follow-up.

## 2. Materials and Methods

### 2.1. Participants

This study represents a follow-up of a wider study that investigated brain structure and function, cognition, and psychological wellbeing in adolescents first diagnosed with ME/CFS and healthy adolescent controls [35]. A total of 48 participants (25 with ME/CFS and 23 healthy controls) participated in the original study. Inclusion and exclusion criteria for this study have been described in detail previously [35]. Participants included adolescents aged 13–18 years diagnosed with ME/CFS by a paediatrician specialising in ME/CFS at an Australian tertiary children’s hospital using the Canadian Criteria adapted for paediatrics [36,37] and healthy adolescent controls aged 13–18 with no history of ME/CFS or other chronic illnesses. Exclusion criteria at study enrolment were insufficient English to complete the questionnaires, major depression or anxiety disorder, history of psychosis or bipolar disorder, pre-existing developmental disability or brain injury, and current use of any medication that may affect brain function.

All 48 participants were invited to participate in the follow-up study approximately two years after their participation in the original study when they were first diagnosed with ME/CFS. Ten participants (7 ME/CFS and 3 controls) could not be contacted despite multiple attempts, and 4 participants withdrew at follow-up (1 ME/CFS and 3 controls). Therefore, 34 participants (17 adolescents originally diagnosed with ME/CFS and 17 healthy controls) took part in both the original and follow-up studies and were included as part of the current investigation. Informed consent was obtained from all participants and their parents, and no compensation or incentives were offered to participate in the research. The study was approved by The Royal Children’s Hospital Human Research Ethics Committee (HREC 32233, 37200).

### 2.2. Procedure

*Original study at diagnosis (Baseline).* Participants completed standardised questionnaires via REDCap Software (version 5.10.2, Vanderbilt University, Nashville, TN, USA, 2014; [38]). The questionnaires aimed to assess factors associated with health and psychological wellbeing, namely fatigue, sleep quality and hygiene, pain, anxiety, depression, and health-related quality of life. Questions regarding demographic characteristics were also completed, and for the ME/CFS cohort, additional clinical information was collected by their paediatrician in consultation with the family. This included illness characteristics such as time from symptom onset to diagnosis (study enrolment) (i.e., how long had symptoms been present when diagnosis was made) and perceived illness trigger, as well as diagnostic symptom criteria.

*Follow-up study.* Participants completed the same questionnaires administered at baseline. In addition, the adolescents originally diagnosed with ME/CFS were also asked to complete a health questionnaire about symptoms experienced over the past 3 months.

### 2.3. Measures

Health and psychological wellbeing measures across five domains were collected at both baseline and follow-up, and shown in Table 1. These were validated for use in children, adolescents and young people up to 25 years of age, and demonstrated good to excellent reliability, validity and internal consistency in adolescents with ME/CFS, other chronic health conditions, and healthy adolescents [8,29,39,40,41,42,43,44,45,46].

A short researcher-designed health questionnaire for the ME/CFS cohort was administered at baseline and follow-up, based on the diagnostic criteria for the paediatric case definition of ME/CFS and developed by the Pediatric ME/CFS Case Definition Working Group [36,37,52]. At baseline, the health questionnaire was completed by the ME/CFS patient’s paediatrician who specialised in ME/CFS, and at follow-up the questionnaire completed by the adolescent with ME/CFS (some words were rephrased to be understood by a younger audience, see Supplementary Table S1 for comparison).

Two main measures were obtained from the health questionnaire: (a) fulfilment of ME/CFS diagnostic criteria (including ‘severe’, ‘moderate’, or ‘atypical’ ME/CFS, [36,37,53], and (b) whether patients subjectively perceived they had ME/CFS at follow-up (‘Do you still have ME/CFS? Yes or No.’). As defined in the paediatric case definition [37], ‘severe ME/CFS’ participants had to meet all six classic symptom criteria, including at least one symptom from two of the three categories of autonomic, neuroendocrine, and immune manifestations. ‘Moderate ME/CFS’ participants were defined as meeting five out of the six classic symptom criteria, including at least one symptom in any of the three autonomic, neuroendocrine, and immune categories. ‘Atypical ME/CFS’ participants were defined as meeting two to four of the classic six symptom categories. At follow-up, the questionnaire relied on self-report rather than medical consultation and examination with their clinician and, as such, the case definition criteria for exclusionary conditions and concomitant disorders and ratings of severity were not included. At follow-up, the questionnaire also asked about the types of health professional/s seen for management of the participant’s condition, the number of visits to that/those health professionals since baseline, and the impact of their ME/CFS on their participation in school, university or employment (‘a lot’, ‘a little’, or ‘not at all’).

Finally, participants completed the *Wechsler Abbreviated Scale of Intelligence—Second Edition (WASI-II): Two-subtest Full Scale Intellectual Quotient* (Vocabulary and Matrix Reasoning subtests) at baseline and follow-up to obtain an estimation of their general intellectual ability, or FSIQ [54]. Standardised scores were reported (*M* = 100, *SD* = 15).

### 2.4. Statistical Analysis

All data were analysed using the statistical analysis program Stata 16.0 (StataCorp Release 16, College Station, TX, USA: StataCorp LLC, 2019), and screened for violations of statistical assumptions. The sample characteristics were summarised using descriptive statistics. Independent samples *t*-tests and chi-square tests were used to assess group differences at baseline and follow-up.

For the first aim, analysis of group differences in aspects of health and psychological wellbeing over time involved a single linear mixed-effects regression model for each outcome (dependent variable). Models included time (baseline vs. follow-up) and group (ME/CFS vs. control) as predictors, an interaction term between group and time, a random intercept for each participant to allow for clustering of observations within a participant, and follow-up time interval in years as a covariate (i.e., time since participation in original study). The linear mixed-effects regression results were presented as estimated mean differences (fixed main effects and pairwise contrasts of the dependent variable); that is, unstandardised regression coefficients (*b*) with their 95% confidence intervals (CIs), and associated standard errors (SE). A significance level of 0.05 was used for all models, and rather than relying solely on *p* values, Cohen’s *d* was calculated to determine the magnitude of the effect and interpreted according to Cohen’s [55] and Sawilowsky’s [56] guidelines (0.20 and below = small, 0.50 = moderate, 0.80 = large, 1.20 and above = very large). Moderate to large values were considered clinically meaningful.

For the second aim, frequency statistics and percentages were used to summarise participants’ responses to the health questionnaire; namely, (a) the proportion that fulfilled paediatric ME/CFS diagnostic criteria [36,37], (b) frequency of reported ME/CFS symptoms, and (c) the proportion who perceived they still had ME/CFS at follow-up. Responses were dummy coded (1 = met criteria; 0 = did not meet criteria) and then summed, with a possible total score range of 0–6, to reflect the classic six paediatric ME/CFS case definition criteria.

For the third aim, multiple logistic mixed-effects regression models were performed to determine which baseline variables of health and psychological wellbeing best predicted fulfilment of ME/CFS diagnostic criteria at follow up (controlling for time interval between studies), via unstandardised regression coefficients, 95% CIs, and *p* values. ORs were used as the magnitude of the effect and were interpreted according to Rosenthal’s [57] guidelines (1.5:1 = small, 2.5:1 = moderate, 4:1 = large, 10:1 = very large). Pearson correlations were also used to assess the strength and direction of the linear associations between the baseline and follow-up variables of health and psychological wellbeing and fulfilment of ME/CFS criteria at follow-up.

## 3. Results

### 3.1. Participant Characteristics

As shown in Table 2, there were no significant group differences in mean age, sex (proportion of females), socio-economic status, or FSIQ. Results remained unchanged when the analysis was repeated for participants at baseline who were lost to follow-up (ME/CFS group: n = 17 participated at follow-up, n = 8 lost to follow-up; Control group: n = 17 participated at follow-up, n = 6 lost to follow-up). Average time interval between baseline and follow-up was significantly longer for adolescents with ME/CFS compared with controls, so follow-up time interval was included as a covariate in subsequent mixed-effects regression analyses.

### 3.2. Group Differences in Trajectories of Health and Psychological Wellbeing from Baseline to Follow-Up

**Figure 1:**
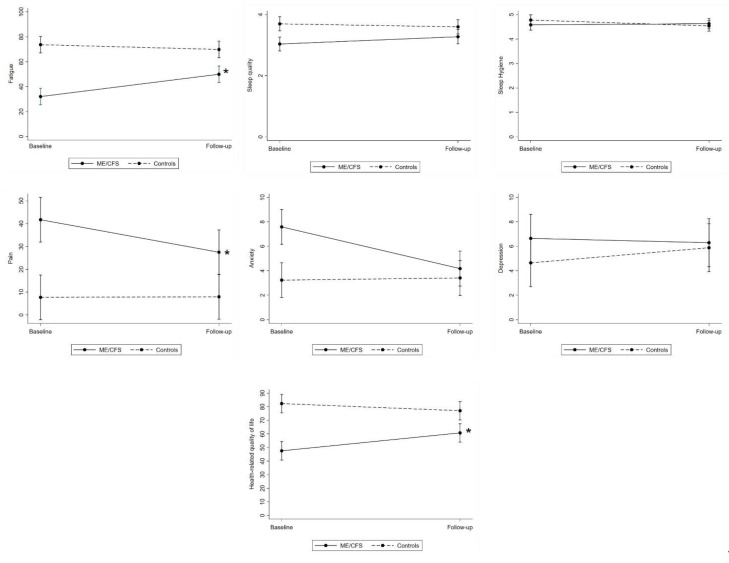
Estimated mean differences in measures of health and psychological wellbeing over time (baseline vs. follow-up) and between groups (ME/CFS vs controls). * Significant within-group change over time at 0.05 level and moderate to large Cohen’s d effect sizes ≥ 0.5.

Estimated mean group differences (ME/CFS vs. control) over the approximate two-year period (baseline vs. follow-up) are shown in Table 3 and Figure 1. Raw means for each measure can be found in Table S2.

Fatigue. A significant main effect of group, time, and group by time interaction effect was observed. At baseline, the ME/CFS group had a significantly greater level of problems related to fatigue than controls (mean difference = 41.62, *p* < 0.001, *d* = 1.47). This group difference diminished over time, with the ME/CFS group reporting significant improvement in fatigue levels from baseline to follow-up (mean difference = 17.89, *p* < 0.001, *d* = 0.66), and the control group remaining relatively stable (mean difference = −3.84, *p* > 0.05, *d* = 0.14). At follow-up, the ME/CFS group still reported significantly greater fatigue than controls, although the magnitude of this effect was reduced compared to baseline (mean difference = 19.89, *p* < 0.001; *d* = 0.70).

Sleep quality. A significant main effect of group, time, and group by time interaction effect was observed. At baseline, the ME/CFS group reported significantly poorer sleep quality than controls (mean difference = 0.66, *p* <0.001, *d* = 0.67). This magnitude of change over time in sleep quality differed between the groups, with the ME/CFS group reporting significant improvement from baseline to follow-up (mean difference = 0.24, *p* = 0.03, *d* = 0.37), and the control group remaining stable (mean difference = −0.10, *p* > 0.05, *d* = 0.15). At follow-up, there was no significant difference in sleep quality between the two groups (mean difference = 0.32, *p* >0.05, *d* = 0.33).

Sleep hygiene. A significant time by group interaction effect was observed, however the individual effects of time and group were very small and did not reach significance. The groups showed similar levels of sleep hygiene at both baseline and follow-up, and the interaction effect appeared to be driven by a small decline in sleep hygiene in the control group over time (mean difference = −0.24, *p* = 0.01, *d* = 0.44).

Pain. A significant effect of group, time, and group by time interaction effect was observed. At baseline, the ME/CFS group reported significantly greater severity of present pain than controls (mean difference = −33.97, *p* < 0.001, *d* = 0.81). Over time, the ME/CFS group reported a significant decline in pain from baseline to follow-up (mean difference = −14.24, *p* < 0.01, *d* = 0.48), and the control group remained stable (mean difference = 0.24, *p* > 0.05, d = 0.01). At follow-up, the ME/CFS group continued to report significantly greater pain than controls, although the magnitude of this effect was reduced compared to baseline (mean difference = −19.50, *p* < 0.01; *d* = 0.46).

Anxiety. A significant main effect of group, time, and group by time interaction effect was observed. At baseline, the ME/CFS group reported significantly greater levels of anxiety than the control group (mean difference = −4.35, *p* < 0.001, *d* = 0.71). Over time, anxiety levels significantly decreased for the ME/CFS group (mean difference = −3.41, *p* < 0.001, *d* = 0.82), but did not significantly change for controls (mean difference = −0.18, *p* > 0.05, *d* = 0.04), such that at follow-up there was no significant difference in anxiety levels between the two groups (mean difference = −0.76, *p* > 0.05, *d* = 0.12).

Depression. No significant main effects of time or group, nor a significant time by group interaction effect was observed (all *p* > 0.05). Mean group differences in depression from baseline to follow-up were associated with negligible effect sizes for both groups (both *p* > 0.05 and small d).

HRQOL. A significant main effect of group, time, and group by time interaction effect was observed. At baseline, the ME/CFS group reported significantly poorer HRQOL than controls (mean difference = 34.75, *p* < 0.001, *d* = 1.19). Over time, this magnitude of this group difference diminished, whereby HRQOL significantly improved for the ME/CFS group (mean difference = 13.19, *p* < 0.001, *d* = 0.66) but did not significantly change for the control group (mean difference = −5.24, *p* > 0.05, *d* = 0.26). At follow-up, the ME/CFS group still reported significantly worse HRQOL than controls, although the magnitude of the effect was reduced compared to baseline (mean difference = 16.32, *p* = 0.001, *d* = 0.56).

### 3.3. ME/CFS Symptomatology and Fulfilment of ME/CFS Diagnostic Criteria

At baseline, all 17 adolescents diagnosed with ME/CFS by their consultant ME/CFS specialist paediatrician fulfilled criteria for ME/CFS (‘severe ME/CFS’: 59%, *n* = 10; ‘moderate ME/CFS’: 41%, *n* = 7). At follow-up, 65% (*n* = 11) of participants fulfilled criteria for ME/CFS (‘severe ME/CFS’: 24%, n = 4; ‘moderate ME/CFS’: 18%, *n* = 3; ‘atypical ME/CFS’: 24%, *n* = 4). Of the 4 participants who met criteria for ‘atypical ME/CFS’ (i.e., only requiring 2 to 4 symptoms be endorsed) at follow-up, all 4 met criteria for the classic criteria of fatigue, sleep problems, and pain. Six of the 17 (35%) did not fulfil ME/CFS criteria at follow-up, and none of these endorsed persistent and unrelenting fatigue as a symptom.

Of the majority that self-reported as having ME/CFS at follow-up in response to the question ‘do you still have ME/CFS?’ (82%, *n* = 14), a greater proportion fulfilled criteria for ME/CFS than not (79% vs. 21%, respectively). Nine of the 14 participants reported that their ME/CFS had impacted ‘a lot’ on their participation in school, studies or employment, while 5 reported that it impacted ‘a little’. All three participants who self-reported as not having ME/CFS at follow-up did not fulfill criteria for ME/CFS. Breakdown of participants who fulfilled paediatric ME/CFS diagnostic criteria at both time points is summarised in Table S3.

**Figure 2:**
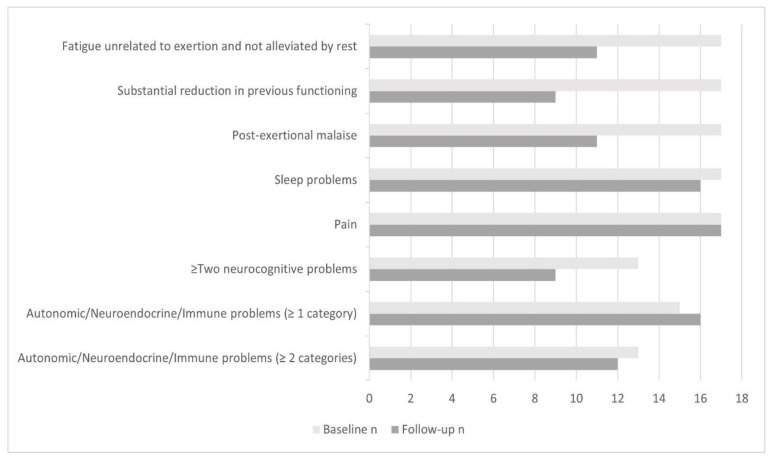
The number of participants who fulfilled each of the classic criteria for paediatric ME/CFS [36,37] for 3 or more months at baseline and follow-up (*n* = 17):(1) unexplained, persistent fatigue that is unrelated to exertion and not alleviated by rest, and represents a substantial reduction in previous functioning (criterion 1A, B, C), (2) post-exertional malaise (criterion 2), (3) sleep problems (criterion 3), (4) pain (criterion 4), (5) two or more neurocognitive problems (criterion 5), and (6) autonomic, immune, and/or neuroendocrine problems (criterion 6).

Figure 2 shows the number of participants who fulfilled each of the classic symptom criteria for paediatric ME/CFS at baseline and follow-up (*n* = 17). Pain was the most endorsed symptom at follow-up (100%), closely followed by sleep problems (94.1%) and one or more autonomic/neuroendocrine/immune problems (94.1%).

### 3.4. Predictors of ME/CFS Criteria Fulfilment at Follow-Up

None of the baseline variables of health and psychological wellbeing were significant predictors of fulfilment of ME/CFS diagnostic criteria at follow-up (all *p* > 0.05, d range = 0.02–0.33, OR range = 0.83–2.54). Pain experienced at baseline was close to reaching significance (*b* = 0.05, *p* = 0.053) and was associated with a moderate effect size (*d* = 0.33), but a small OR (OR = 1.05; SE(OR) = 0.02). For the Pearson correlation analysis, only pain experienced at baseline was significantly associated with ME/CFS criteria fulfilment at follow-up, with moderate effect (*R* = 0.6, *p* = 0.02). The Pearson correlation matrix for this analysis can be found in Table S4.

## 4. Discussion

This study aimed to examine trajectories of health and psychological wellbeing across an (approximately) two-year period in adolescents diagnosed with ME/CFS compared with their healthy peers. It also aimed to track patients’ ME/CFS symptomatology and fulfillment of paediatric ME/CFS diagnostic criteria at follow-up, and determine whether ME/CFS criteria fulfilment at follow-up could be predicted by aspects of health and psychological wellbeing at diagnosis.

### 4.1. Trajectories of Health and Psychological Wellbeing from Baseline to Follow-Up

Greater levels of fatigue, pain, and anxiety, and lower levels of sleep quality and HRQOL were observed amongst adolescents with ME/CFS at baseline compared with their healthy peers. This is consistent with previous cross-sectional findings in adolescents with ME/CFS from our own team [3,9,43,58] and others [7,13,59,60,61,62,63,64]. This is perhaps unsurprising given that the cohort met paediatric case definition criteria for either severe (59%) or moderate (41%) severity ME/CFS at baseline, and the assessed domains of fatigue, pain, and sleep problems map onto the known clinical symptoms experienced in this condition. Importantly, the group disparity in these features of health and psychological wellbeing became less pronounced from diagnosis to follow-up, due to the significant improvement observed over time in the ME/CFS group, and the relative stability of the control group over time. Indeed, improvement in ME/CFS patient-reported outcomes have previously been shown in the domain of fatigue [12,65], with a recent systematic review showing recovery rates for paediatric ME/CFS of between 15% and 85% based on outcome measures of fatigue severity [30]. Fatigue and HRQOL have also been shown to co-vary in paediatric ME/CFS [43], which may account for the relative improvement being observed in both these domains over the follow-up period. Trends for a decline in the presence of pain or sleep disturbance over the follow-up period has also been noted in paediatric ME/CFS, regardless of intervention received [12]. The added value of the current study is that we were able to assess levels of severity within ME/CFS symptom domains using the same measures at baseline and follow-up. This allowed for a more comprehensive evaluation of change over time in comparison with healthy controls.

A positive finding from this study was that with significant improvement over time, adolescents with ME/CFS became comparable to their healthy peers at follow-up in their level of anxiety and sleep quality. The lack of follow-up studies assessing anxiety in paediatric ME/CFS make the reasons for this improvement in anxiety level unclear. It is possible that anxiety experienced at baseline related to the diagnosis itself and/or diagnostic and prognostic uncertainty, all of which may have reduced in impact over time. Gradual acceptance and better management of their chronic condition may also have played a role in improving anxiety and sleep quality, including multi-disciplinary input in the post-diagnosis period. Certainly, many participants with ME/CFS continued to be managed by a general practitioner (47% of the ME/CFS cohort), paediatrician (41%), psychologist (29%) and/or physiotherapist (29%) for more than 3 visits (71%) over the course of the study period. Inter-relatedness of the domains studied would also suggest that management, rehabilitation and improvement in one area would likely lead to symptom reduction in related domains (i.e., fatigue severity and anxiety have been shown to co-vary in adolescents with ME/CFS [7,32]).

More concerning was the finding that despite significant and clinically-meaningful improvement over time (with moderate to large effect sizes), adolescents with ME/CFS continued to show significantly greater fatigue, pain and poorer health-related quality of life than their healthy peers at follow-up. This observation is important for the treating clinician to understand so that a more realistic prognosis and need for intervention can be discussed with the young person and their family at the initial diagnosis and start of treatment. Van Geelen et al. [26] found considerable levels of fatigue in adolescents with ME/CFS at a similar follow-up timeframe to the current study (approximately 2 years), despite substantial health care use in the cohort, and this correlated with greater pain and poorer health and psychological wellbeing. We recognise that the patient cohorts from our (and Van Geelen’s) study were comparatively early in their trajectory of recovery, with further improvement expected over time. Rowe [11] reported a mean paediatric ME/CFS illness duration of 5 years for those reporting recovery, but with a range of 1 to 15 years. In the current cohort, the mean two-year follow-up time interval from diagnosis had a range of 1 to 4 years, with the onset of symptoms prior to diagnosis ranging from 3 months to over 24 months. Our cohort may also have represented ME/CFS cases of greater severity and reduced functioning given they had been referred for specialist tertiary care.

Unlike previous studies [6,31,62,66], we did not find higher rates of depression in adolescents with ME/CFS compared with healthy controls, nor an increase in depression over time. However, our findings do support two recent paediatric ME/CFS follow-up studies showing stable depression levels over time [67,68]. Although Loades et al. [68] observed consistently higher levels of depression in the paediatric ME/CFS group than healthy controls, baseline ME/CFS depression was found to explain most of the variance in follow-up ME/CFS depression, which appears to suggest stable depression levels across time in paediatric ME/CFS. Of note, Loades et al.’s sample included a higher proportion of adolescents with depression compared with previous studies [6,66]. It may be that there is a subtype of ME/CFS that is particularly associated with comorbid depression [69], which was represented in Loades et al.’s [68] sample but not in our study. Major psychiatric illness that could adequately explain fatigue symptoms was an exclusionary criteria in our study, which would have played an additional role.

### 4.2. Fulfilment of Paediatric ME/CFS Diagnostic Criteria at Follow-Up

The main finding from the second aim of our study was that approximately two thirds (65%) of participants continued to fulfill paediatric ME/CFS diagnostic criteria at follow-up, which included cases of severe, moderate, and atypical severity. The remaining third (35%) were not classified as meeting criteria and more likely reflected a sub-clinical sample of individuals that had improved considerably since diagnosis, given that none reported unexplained, persistent fatigue that represented a substantial reduction in previous functioning. This 35% of participants could be interpreted as having improved clinical status since diagnosis, which would fall within the observed ‘recovery’ range in Moore et al.’s [30] systematic review of paediatric ME/CFS longitudinal studies (ranging from 5% to 83% recovery). However, there are obvious limitations in inferring recovery and when comparing results across these follow-up studies, given the variability in case definition, inclusion criteria, and definitions of recovery used. In the current study, follow-up diagnostic status was determined through self-report only. The known fluctuating nature and severity of ME/CFS experienced by patients, as well as our lack of diagnostic biomarker/s, also makes it more difficult to establish accurate diagnostic status at follow-up. This is supported by Rowe [11] who found 58% of paediatric ME/CFS patients had a fluctuating severity pattern of illness over the follow-up period, with 14% reporting a consistent level of severity, and 12% showing a relapsing and remitting pattern.

Consistent with the majority of patients fulfilling paediatric ME/CFS criteria at follow-up, the majority of patients (82%) self-identified as continuing to have the condition at follow-up. Of these patients, 79% did indeed fulfil criteria for ME/CFS, while 21% did not. On one hand, the disparity could be an issue of construct validity. Whilst the Canadian consensus criteria for paediatric ME/CFS is considered an improvement upon previous case definitions [70], greater sensitivity and specificity may be needed given the heterogeneity of the illness [71,72]. On the other hand, a disparity between adolescent-defined and criteria-defined clinical status could be a function of the dimensional nature of many ME/CFS symptoms, including pain, sleep problems and fatigue [73,74]. It is possible that adolescents interpret the persistence of their somatic symptoms as a sign of overall ME/CFS persistence, even if the more cardinal ME/CFS symptoms (e.g., post-exertional malaise) are no longer an issue and/or the severity of their somatic symptoms has lessened. This may be supported by the finding that pain was the most endorsed symptom at follow-up in the ME/CFS group (100% of the cohort). Whatever the cause, if there is a disparity between the subjective experience of paediatric ME/CFS and what is being captured by current criteria, this has ramifications for research and practice. It is recommended that future research increase the specificity of ME/CFS somatic symptom criteria by drawing upon up-to-date research in sleep, fatigue and pain, in addition to continuing to dedicate resources towards identifying diagnostic biomarkers for paediatric ME/CFS.

### 4.3. Predictors of ME/CFS Criteria Fulfilment

The main finding from the third aim of our study was that no aspects of baseline health and psychological wellbeing were found to significantly predict ME/CFS criteria fulfilment at follow-up, with any great effect. However, despite not reaching significance as a predictor (with moderate effect), pain at baseline was significantly and positively associated with meeting criteria at follow-up, and was also the most commonly endorsed symptom by patients at follow-up. This would suggest that the experience of pain early in illness course may be relevant to later diagnostic status in the wider paediatric ME/CFS population, which is supported by previous research [3,75].

Although many reports imply that the presence of poor health at ME/CFS onset influences future recovery [1,63,76], empirical support is lacking. In fact, investigations focused on anxiety and depression suggest otherwise. For example, Rowe [11] reported no association between baseline depression/anxiety and recovery, and whilst Rimes et al. [15] found an association between baseline anxiety/depression and *new onset* chronic fatigue, they found no association between baseline anxiety/depression and *persistent* chronic fatigue. Rowe [11] and Rimes et al. [15] findings are consistent with the present study’s results. It is worth noting that previous follow-up studies have tended to identify demographic predictors (rather than health/psychological predictors) of future clinical status, namely: older age, female gender, higher IQ, higher BMI, and school absenteeism [12,15,24,76]. Those studies that *have* found associations between baseline health/psychological wellbeing—e.g., sleep quality [77], depression [28,78], anxiety and fatigue [32]—and follow-up recovery status have relied on a proxy for ME/CFS (e.g., “CFS-like symptoms”, “Chronic Disabling Fatigue”, etc.). Therefore, it remains to be seen whether baseline aspects of health and psychological wellbeing are useful prognostic indicators of future diagnostic status or recovery in adolescents who have been diagnosed by a paediatric ME/CFS specialist.

### 4.4. Study Limitations and Strengths

It is important to acknowledge the possibility that those adolescents with ME/CFS who were lost to follow-up (*n* = 8) may potentially have been more functionally impaired than those who participated at follow-up, thereby influencing the representativeness of our sample. Importantly, analysis comparing baseline data for those involved in and those lost to follow-up revealed no significant differences in demographic characteristics (age, SEIFA, sex) or intellectual ability (FSIQ), which suggests these influences were minimal. Our study would also have benefited from the inclusion of a comparison group such as adolescents with fibromyalgia, and with a larger sample size, would have been powered to separate the cohort into subgroups of mild, moderate, and severe ME/CFS for analysis. This would have helped to offset any influence of regression toward the mean when evaluating change over time [79], and helped to improve our ability to detect significant associations between baseline health status and ME/CFS criteria fulfilment at follow-up. Given these considerations, caution should be taken when generalising the present study’s findings to adolescents with severe ME/CFS or sufficiently poor health at diagnosis, with the view that they may overestimate improvement in long-term outcomes for this group. In addition, further information regarding types of medication and treatments over the course of the follow-up period would be beneficial for future evaluation of the factors that may influence longer-term outcomes in this population.

A key strength of this study was the use of a case-controlled, longitudinal design and a cohort of well-characterised adolescents with ME/CFS whose diagnosis was confirmed by a specialist paediatrician using consensus clinical criteria, and whose follow-up diagnostic status was recorded using the same criteria. While it is acknowledged that some diagnostic items assessed at follow-up had to be re-phrased for the younger patient audience, the content at both timepoints was the same (as seen in Supplementary Table S1) allowing for reasonable comparison across time. This is an improvement upon previous follow-up studies which have relied on a proxy for ME/CFS diagnosis [32,78,80] or have not included a control group for comparison [12,24,25]. Using the same measures at baseline and follow-up also allowed for direct comparisons over time, including measures of health and psychological wellbeing which are less often studied in longitudinal paediatric ME/CFS studies. To the authors’ knowledge, this study represents one of the first case-controlled follow-up studies of adolescents with ME/CFS in terms of their health, well-being and longer-term prognosis of their condition.

### 4.5. Clinical Implications and Future Directions

Given the observed persistence of ME/CFS symptoms, poorer health and reduced psychological well-being at follow-up compared with healthy controls (i.e., fatigue, pain, and health-related quality of life problems), the current study highlights the need for early identification and targeted and intensive treatment in these domains that continues at least two years post-diagnosis, but ideally longer. The symptom domain of pain may be a particularly pertinent area of focus in the management of paediatric ME/CFS, given pain was most frequently endorsed at follow-up, and pain at baseline was significantly associated with fulfilment of ME/CFS criteria at follow-up. Clinical multidisciplinary strategies targeting pain relief and management including medication, physiotherapy, cognitive and behavioural techniques (i.e., meditation, mindfulness and acceptance and commitment therapy), and regular follow-up with the treating physician will be essential in this regard. The use of standardised and consistent measurement of symptomatology across illness course, including evidence-based consensus criteria, and person-centred measurements of recovery will also be important in tracking patients’ clinical outcomes and wellbeing over time in a meaningful way. Based on our findings, future research must employ multiple follow-up time points over a longer time period (i.e., longer than 4 years) to accommodate and record the fluctuating nature of symptoms and illness severity over the ME/CFS illness course. Such information would be invaluable for understanding the different types of illness trajectories experienced by patients, and best predictors of recovery. It would also help clinicians, adolescent patients, and their families in preparing young people with ME/CFS for the potential transition from family-oriented paediatric health services to more independently-oriented adult health services if required.

## Figures and Tables

**Table 1:** Measures to evaluate health and psychological wellbeing in adolescents with ME/CFS and healthy controls.

Measure Domain	Name of Measure	Description of Measure
Fatigue	PedsQL™ Multidimensional Fatigue Scale [47,48]	18-item Likert-rated scale (from ‘Never’ or 0 to ‘Almost always’ or 4) that assesses level of subjective fatigue over the past month. Items reversed scored, linearly transformed to a 0–100 scale, and summed over the number of items answered to form a Total Fatigue score. Higher total fatigue scores reflected fewer problems related to fatigue.
Sleep quality and sleep hygiene	Adolescent Sleep Wake Scale (ASWS) and Adolescent Sleep Hygiene Scale (ASHS) [49]	Two 28-item instruments that assess aspects of sleep over past month: ASWS assesses subjective sleep quality including evaluation of sleep initiation and maintenance; ASHS assesses sleep hygiene and sleep practices. Items measured on a 6-point Likert scale (1 = always; 6 = never). Higher total scores indicate better sleep quality and hygiene.
Pain	PedsQL™ Pediatric Pain Questionnaire Visual Analogue Scale [50]	Self-rated 100 mm scale to measure intensity of present pain, from ‘not hurting’ or ‘no pain’ (0) to ‘hurting a whole lot’ or ‘severe pain’ (100).
Depression and Anxiety	Hospital Anxiety and Depression Scale [51]	Consists of 14 items (7 in each subscale) and each item is scored from 0 to 3. Higher total scores indicate greater levels of depression and anxiety.
Health-related quality of life (HRQOL)	PedsQL™ Core Generic Module [46,47]	Widely-used measure of health-related quality of life (HRQOL) assessing subjective impact of health status on wellbeing and life satisfaction. Respondents rate 23 items on 5-point Likert scale (0 = never a problem; 4 = almost always a problem) according to how much of a problem each item has been over the previous month. Items reversed scored and linearly transformed to create a total score ranging between 0 and 100. Higher total scores indicate better perceived HRQOL.

**Table 2:** Participant characteristics at baseline and follow-up.

Participant Characteristics	ME/CFS (*n* = 17)	Controls (*n* = 17)	Independent *t*-test	*p*-Value
Age in years ((M (SD; range))				
Baseline	15.99 (1.59; 13.42–18.92)	15.90 (1.60; 13.33–18.08)	0.17	0.86
Follow-up	18.78 (1.63; 15.5–21.58)	18.20 (1.56; 15.58–20.58)	1.07	0.29
Female sex (%, n)	82%, 14	65%, 11	Χ^2^ = 1.36	0.24
Socio-economic Indexes for Areas (SEIFA) (M (range)) *	7.12 (1–10)	7.81 (1–10)	−0.73	0.47
Follow-up time interval in years (M (SD; range))	2.75 (0.81; 1.83–4.58)	2.27 (0.43; 1.67–3)	2.14	0.04
Estimated FSIQ (M (SD; range)) **				
Baseline	103.75 (13.67; 86–145)	107.71 (12.50; 89–130)	−0.87	0.39
Follow-up	105.56 (11.41; 90–136)	109.94 (12.98; 81–129)	−1.03	0.31
Time from symptom onset to diagnosis (study enrolment) (%, *n*)		-	-	-
3–6 months	24%, 4	-	-	-
7–12 months	29%, 5	-	-	-
13–24 months	24%, 4	-	-	-
>24 months	24%, 4	-	-	-
Perceived illness trigger at study enrolment (%, *n*) ** ^×^				
Infectious Illness	41%, 7	-	-	-
Accident	12%, 2	-	-	-
Severe stress	12%, 2	-	-	-
Immunisation	6%, 1	-	-	-
Trip or vacation	0%, 0	-	-	-
No identifiable trigger	24%, 4	-	-	-
Visited health professional or specialist between baseline and follow-up ^×^				
No	24%, 4			
Yes	76%, 13			
General Practitioner	47%, 8			
Paediatrician	41%, 7			
Physiotherapist	29%, 5			
Psychologist	29%, 5			
Cardiologist	12%, 2			
Gynaecologist	12%, 2			
Psychiatrist	6%, 1			
Neurologist	6%, 1			
Gastroenterologist	6%, 1			
Naturopath	6%, 1			
Number of visits to that health professional or specialist between baseline and follow-up				
0 visits	24%, 4			
1 visit	0%, 0			
2 visits	12%, 2			
3 visits	0%, 0			
>3 visits	65%, 11			

* Control *n* = 16; ** ME/CFS *n* = 16; ^×^ Participants reported more than one trigger. For participant characteristics for the full cohort of 48 adolescents (25 ME/CFS, 23 controls) that participated in the original study, see [35].

**Table 3:** Estimated mean differences in health and psychological wellbeing over time (baseline vs. follow-up) and between groups (ME/CFS vs. Control).

Measures of Health and Psychological Wellbeing	Estimated Mean Difference (*b*) with 95% CIs	SE	*p*-Value	Effect Size (Cohen’s *d)*
Fatigue				
Time	17.89 (8.83, 26.96)	4.62	<0.001	0.66
Group	41.62 (32.10, 51.14)	4.86	<0.001	1.47
Time × Group	−21.73 (−34.55, −8.91)	6.54	0.001	0.57
Sleep quality				
Time	0.24 (0.02, 0.45)	0.11	0.03	0.37
Group	0.66 (0.33, 0.99)	0.17	<0.001	0.67
Time × Group	−0.34 (−0.64, −0.03)	0.16	0.03	0.37
Sleep hygiene				
Time	0.05 (−0.13, 0.23)	0.09	0.60	0.09
Group	0.20 (−0.11, 0.50)	0.16	0.20	0.22
Time × Group	−0.29 (−0.55, −0.03)	0.13	0.03	0.38
Pain				
Time	−14.24 (−24.23, −4.24)	5.10	<0.01	0.48
Group	−33.97 (−48.14, −19.80)	7.23	<0.001	0.81
Time × Group	14.57 (0.33, 28.61)	7.21	0.045	0.34
Anxiety				
Time	−3.41 (−4.81, −2.02)	0.71	<0.001	0.82
Group	−4.35 (−6.42, −2.29)	1.05	<0.001	0.71
Time × Group	3.59 (1.62, 5.56)	1.01	<0.001	0.61
Depression				
Time	−0.35 (−2.18, 1.48)	0.93	0.71	0.06
Group	−2.00 (−4.84, 0.84)	1.45	0.17	0.24
Time × Group	1.59 (−1.00, 4.18)	1.32	0.23	0.21
HRQOL				
Time	13.19 (6.48, 19.91)	3.43	<0.001	0.66
Group	34.75 (24.95, 44.55)	5.00	<0.001	1.19
Time × Group	−18.43 (−27.93, −8.94)	4.84	<0.001	0.65

## Data Availability

The data presented in this study are available in text and the supplementary material, in Josev et al. (2020), and on request from the corresponding author, and are not publicly available due to patient privacy and ethical restrictions.

## Supplementary Materials

The following are available online at https://www.mdpi.com/article/10.3390/jcm10163603/s1, Supplementary Table S1: Classic ME/CFS diagnostic criteria of the Canadian Consensus Criteria adapted for pediatrics (left column), and how these were assessed at baseline (middle column) and follow-up (right column), Table S2: Raw means of measures of health and psychological wellbeing at baseline and follow-up for adolescents with ME/CFS and healthy controls, Table S3: Participants who fulfilled paediatric ME/CFS diagnostic criteria at baseline and follow-up, Table S4: Pearson correlation matrix between health and psychological wellbeing measures and fulfilment of paediatric ME/CFS diagnostic criteria
