# Supplementary material for: Health, Wellbeing, and Prognosis of Australian Adolescents with Myalgic Encephalomyelitis/Chronic Fatigue Syndrome (ME/CFS): A Case-Controlled Follow-Up Study
Source: J Clin Med. 2021 Aug 16;10(16):3603. doi: 10.3390/jcm10163603 (PMC8396969; doi:10.3390/jcm10163603)
Supplement: Supplementary file 1 [file jcm-10-03603-s001.zip › Josev_Supplementary Table S1.pdf]

Journal of
Clinical Medicine

MDPI

Supplementary Table S1. Classic ME/CFS diagnostic criteria of the Canadian Consensus Criteria adapted for pediatrics (left column), and how these were assessed at baseline (middle column) and follow-up (right column)

Classic criteria for the paediatric ME/CFS diagnosis	Diagnostic items from the clinician health questionnaire at baseline	Diagnostic items from the patient health questionnaire at follow-up
**The concurrent occurrence of the following classic ME/CFS symptoms must be met for a paediatric ME/CFS diagnosis [1-3], and must have persisted or recurred during the past three months of illness.**	**Instructions to treating paediatrician: Please tick the yes/no boxes and the other symptom boxes if the patient you have diagnosed with ME/CFS has had any of the following for the past 3 months or longer.**	**Instructions to ME/CFS patient: Please tick the yes/no boxes and the other symptom boxes if you have had any of the following for the past 3 months or longer.**
1. Clinically evaluated, unexplained, persistent or relapsing chronic fatigue which must persist or reoccur for at least three months that:<br>A. Is not the result of ongoing exertion<br>B. Is not substantially alleviated by rest	1. Fatigue Criteria<br>Clinically evaluated, unexplained, persistent or relapsing chronic fatigue which must persist or reoccur for at least three months, which is not the result of ongoing exertion, nor substantially alleviated by rest<br>☐ Yes<br>☐ No	1. Fatigue Problems<br>Feelings of constant tiredness, not due to exercise, which does not get better after rest<br>☐ Yes<br>☐ No
C. Results in substantial reduction in previous levels of educational, social and personal activities	Substantial reduction in pre-illness activity levels<br>☐ Yes<br>☐ No	Please estimate and tick how much your illness has affected your ability to participate in school/university/employment:<br>☐ A lot<br>☐ A little<br>☐ Not at all

1. Jason, L., et al., *A pediatric case definition for myalgic encephalomyelitis and chronic fatigue syndrome.* Journal of Chronic Fatigue Syndrome, 2006. **13**(2-3): p. 1-44.

2. Post-exertional malaise and/or post-exertional fatigue.	2. Post-exertional malaise criteria<br>Post-exertional malaise/symptom exacerbation<br>☐ Yes<br>☐ No	2. Energy problems<br>Feeling a loss of energy and concentration even after a small amount of effort when doing activities like walking up a flight of stairs, using a computer, or reading a book<br>☐ Yes<br>☐ No
3. Unrefreshing sleep or disturbance of sleep quantity or rhythm disturbance.	3. Sleep criteria<br>☐ Sleep disturbance<br>☐ Unrefreshing sleep<br>☐ Problems falling asleep<br>☐ Frequent waking<br>☐ Need to nap every day	3. Sleep problems<br>☐ Easily awakened during the night<br>☐ Waking up tired<br>☐ Trouble falling asleep<br>☐ Often waking up during the night<br>☐ Need to nap every day
4. Pain (or discomfort) that is often widespread and migratory in nature. At least one symptom from any of the following:<br>A. Myofascial and/or joint pain<br>B. Abdominal and/or head pain	4. Pain criteria<br>☐ Headaches<br>☐ Muscle pain<br>☐ Joint pain<br>☐ Eye pain<br>☐ Chest pain<br>☐ Abdominal pain<br>☐ Generalised pain	4. Pain problems<br>☐ Headaches<br>☐ Muscle pain<br>☐ Joint pain<br>☐ Eye pain<br>☐ Chest pain<br>☐ Stomach pain<br>☐ Pain all over

5. Two or more neurocognitive manifestations (i.e., impaired memory, difficulty focusing)	5. Neurocognitive criteria<br>☐ Problems with memory and new learning<br>☐ Problems with concentration<br>☐ Other cognitive problem	5. Problems with your thinking<br>☐ Problems with your memory and learning new things (i.e., forgetting things)<br>☐ Problems with your concentration (i.e., hard to pay attention or focus during activities like reading, computer/work, watching television)<br>☐ Other worries about thinking abilities
6. At least one symptom from two of the following three categories:<br>1. Autonomic manifestations<br>2. Neuroendocrine manifestations<br>3. Immune manifestations	6. Other manifestations<br><br>Autonomic:<br>☐ Orthostatic intolerance symptoms (e.g.,<br>☐ Palpitations with or without cardiac arrhythmia<br>☐ Dizziness<br>☐ Vasomotor instability<br>☐ Vertigo and/or light-headedness<br>☐ Respiratory irregularities<br>☐ Intestinal or bladder disturbance with or without irritable bowel syndrome or bladder dysfunction<br>☐ Neurally mediated hypotension<br>☐ Postural orthostatic tachycardia syndrome	6. Other problems<br><br><br><br>☐ Heart beating fast<br>☐ Dizziness<br>☐ Fainting<br>☐ Light-headedness or problems with your balance<br>☐ Shortness of breath<br>☐ Upset stomach

2. Jason, L., et al., *The Development of a Revised Canadian Myalgic Encephalomyelitis Chronic Fatigue Syndrome Case Definition.* American Journal of Biochemistry and Biotechnology, 2010. **6**(2).
3. Jason, L., et al., *Examining criteria to diagnose ME/CFS in pediatric samples.* Journal of Behavioral Health and Medicine, 2010. **1**(3): p. 186.

	Neuroendocrine:<br>☐ Loss of thermostatic stability (feeling hot, feeling cold) and heat/cold intolerance<br>☐ Abnormal appetite, weight change<br>☐ Loss of adaptability and tolerance for stress, worsening of symptoms with stress and slow recovery, and emotional lability<br>☐ Hypersensitivity to touch/noise/light	☐ Often feel too hot or too cold<br>☐ Changes in weight and appetite<br>☐ Feel worse than I normally do when I'm stressed<br>☐ Bothered more than normal by bright light, touch and loud noise
	Immune:<br>☐ Recurrent flu-like symptoms and/or general malaise<br>☐ Recurrent sore throat<br>☐ Tender lymph nodes<br>☐ Susceptibility to viruses/illnesses<br>☐ Hypersensitivity to medications and/or chemicals	☐ Body aches<br>☐ Muscles feel weak<br>☐ Get the shakes<br>☐ Often have a sore throat<br>☐ Feel or get sick often<br>☐ When taking medicine or other chemicals, you:<br>☐ Feel sick<br>☐ Vomiting
