# Supplementary material for: Health, Wellbeing, and Prognosis of Australian Adolescents with Myalgic Encephalomyelitis/Chronic Fatigue Syndrome (ME/CFS): A Case-Controlled Follow-Up Study
Source: J Clin Med. 2021 Aug 16;10(16):3603. doi: 10.3390/jcm10163603 (PMC8396969; doi:10.3390/jcm10163603)
Supplement: Supplementary file 1 [file jcm-10-03603-s001.zip › Josev_Supplementary Table S2.pdf]

Journal of
Clinical Medicine

MDPI

Supplemental Table S2: Raw means of measures of health and psychological wellbeing at baseline and follow-up for adolescents with ME/CFS and healthy controls

Measure	ME/CFS (*n* = 17)	Controls (*n* = 17)
Fatigue (M (SD); range)		
Baseline	31.21 (17.81; 6.94-61.11)	74.51 (9.89; 54.17-93.06)
Follow-up	49.10 (15.29; 20.83-77.78)	70.67 (12.90; 45.83-94.44)
Sleep quality (M (SD); range) (ASWS)		
Baseline	3.04 (0.58; 1.99-4.04)	3.69 (0.46; 2.79-5.54)
Follow-up	3.28 (0.44; 2.33-4.05)	3.59 (0.44; 2.68-4.32)
Sleep hygiene (M (SD); range) (ASHS)		
Baseline	4.60 (0.61; 3.50-5.68)	4.75 (0.36; 4.14-5.46)
Follow-up	4.65 (0.42; 3.79-5.36)	4.51 (0.36; 4.0-5.25)
Pain (M (SD); range)		
Baseline	41.88 (30.01; 0-77)	7.53 (10.42; 0-42)
Follow-up	27.65 (24; 0-64)	7.76 (11.26; 0-46)
Anxiety (M (SD); range)		
Baseline	7.76 (4.32; 2-17)	3.06 (2.08; 0-7)
Follow-up	4.35 (2.62; 1-9)	3.24 (2.70; 0-12)
Depression (M (SD); range)		
Baseline	6.53 (5.49; 0-17)	4.76 (2.46; 1-9)
Follow-up	6.18 (4.10; 0-13)	6.00 (3.95; 1-16)
Health-related QoL (M (SD); range)		
Baseline	47.06 (17.65; 17.03-79.06)	82.78 (10.69; 60.78-98.75)
Follow-up	60.25 (16.07; 34.78-82.97)	77.55 (11.85; 56.56-94.57)
