# Supplementary material for: Health, Wellbeing, and Prognosis of Australian Adolescents with Myalgic Encephalomyelitis/Chronic Fatigue Syndrome (ME/CFS): A Case-Controlled Follow-Up Study
Source: J Clin Med. 2021 Aug 16;10(16):3603. doi: 10.3390/jcm10163603 (PMC8396969; doi:10.3390/jcm10163603)
Supplement: Supplementary file 1 [file jcm-10-03603-s001.zip › Josev_Supplementary Table S3.pdf]

Journal of
Clinical Medicine

MDPI

Supplemental Table S3: Participants who fulfilled paediatric ME/CFS diagnostic criteria at baseline and follow-up

**Participants**	**P1**	**P2**	**P3**	**P4**	**P5**	**P6**	**P7**	**P8**	**P9**	**P10**	**P11**	**P12**	**P13**	**P14**	**P15**	**P16**	**P17**	**%, n**
**Baseline**																		
Fulfilled criteria	✓	✓	✓	✓	✓	✓	✓	✓	✓	✓	✓	✓	✓	✓	✓	✓	✓	100%, 17
Severe	✓	✓	✓	✓	✓		✓		✓					✓	✓		✓	59%, 10
Moderate						✓		✓		✓	✓	✓	✓			✓		41%, 7
Atypical																		0%, 0
**Follow-up**																		
Fulfilled criteria	✓	✓	✓		✓	✓	✓	✓		✓		✓	✓				✓	65%, 11
Severe								✓		✓		✓					✓	24%, 4
Moderate		✓			✓								✓					18%, 3
Atypical	✓		✓			✓	✓											24%, 4
Did not fulfil criteria				✓					✓		✓			✓	✓	✓		35%, 6
Self-reported as having ME/CFS	✓	✓	✓	✓	✓	✓	✓	✓		✓		✓	✓		✓	✓	✓	82%, 14

Note: 'Severe' (6 out of 6 criteria), 'moderate' (5 out of 6 criteria), 'atypical' (2-4 criteria) classifications based on the classic six paediatric ME/CFS case definition symptom criteria which must be experienced for more than three months at baseline and follow-up: (1) unexplained, persistent fatigue that is unrelated to exertion and not alleviated by rest, and represents a substantial reduction in previous functioning (criterion 1A, B, C), (2) post-exertional malaise (criterion 2), (3) sleep problems (criterion 3), (4) pain (criterion 4), (5) two or more neurocognitive problems (criterion 5), and (6) autonomic, immune, and/or neuroendocrine problems (criterion 6) [1, 2]

1. Jason, L., et al., *Examining criteria to diagnose ME/CFS in pediatric samples.* Journal of Behavioral Health and Medicine, 2010. **1**(3): p. 186.
2. Jason, L., et al., *The Development of a Revised Canadian Myalgic Encephalomyelitis Chronic Fatigue Syndrome Case Definition.* American Journal of Biochemistry and Biotechnology, 2010. **6**(2).
