# Supplementary material for: Health, Wellbeing, and Prognosis of Australian Adolescents with Myalgic Encephalomyelitis/Chronic Fatigue Syndrome (ME/CFS): A Case-Controlled Follow-Up Study
Source: J Clin Med. 2021 Aug 16;10(16):3603. doi: 10.3390/jcm10163603 (PMC8396969; doi:10.3390/jcm10163603)
Supplement: Supplementary file 1 [file jcm-10-03603-s001.zip › Josev_Supplementary Table S4.pdf]

Journal of Clinical Medicine

Supplemental Table S4: Pearson correlation matrix between health and psychological wellbeing measures and fulfilment of paediatric ME/CFS diagnostic criteria [1, 2].

		Follow-up								Fulfilment of ME/CFS criteria at follow-up
		Fatigue	Sleep quality	Sleep hygiene	Current Pain	Worst Pain	Anxiety	Depression	HRQOL	
Baseline	Fatigue	-0.17	0.11	0.50*	-0.30	-0.28	-0.51*	-0.16	0.17	-0.36
	Sleep quality	-0.11	0.50*	0.61*	-0.61**	-0.38	-0.35	-0.35	0.17	-0.09
	Sleep hygiene	0.36	0.33	0.81***	-0.39	-0.44	-0.52*	-0.69**	0.48	0.19
	Current Pain	-0.10	-0.51	-0.52*	0.53*	0.63**	0.33	-0.17	-0.54*	0.55*
	Worst Pain	-0.22	-0.44	-0.54*	0.58*	0.54*	0.36	0.16	-0.52*	0.31
	Anxiety	-0.05	-0.31	-0.62**	0.66**	0.48	0.54*	0.46	-0.35	0.11
	Depression	-0.09	-0.30	-0.71**	0.62**	0.56*	0.47	0.53*	-0.36	-0.09
	HRQOL	-0.09	0.32	0.67**	-0.48	-0.45	-0.64**	-0.39	0.38	-0.30
Fulfilment of ME/CFS criteria at follow-up		-0.05	-0.34	-0.36	0.05	0.17	0.34	-0.25	-0.35	

*$p < .05$, **$p < .01$, ***$p < .001$; HRQOL = Health-related Quality of Life

1. Jason, L., et al., *Examining criteria to diagnose ME/CFS in pediatric samples.* Journal of Behavioral Health and Medicine, 2010. **1**(3): p. 186.
2. Jason, L., et al., *A pediatric case definition for myalgic encephalomyelitis and chronic fatigue syndrome.* Journal of Chronic Fatigue Syndrome, 2006. **13**(2-3): p. 1-44.
